# Predictors of Intra-Operative and Post-Operative Pain Associated with Routine Dental Procedures in Children: A Systematic Review and Meta-Analysis

**DOI:** 10.3390/dj12010004

**Published:** 2023-12-25

**Authors:** Mohammed A. Alzubaidi, Bernadette K. Drummond, Jianhua Wu, Adam Jones, Jinous F. Tahmassebi, Vishal R. Aggarwal

**Affiliations:** 1 Department of Preventive Dentistry, Faculty of Dentistry, Taif University, P.O. Box 11099, Taif 21944, Saudi Arabia; dr.m.alzubaidi@tudent.org; 2 Faculty of Medicine & Health, School of Dentistry, University of Leeds, Leeds LS2 9JT, UK; b.k.drummond@leeds.ac.uk (B.K.D.); j.h.wu@leeds.ac.uk (J.W.); j.tahmassebi@leeds.ac.uk (J.F.T.)

**Keywords:** predictors, pain, intra-operative pain, post-operative pain, paediatric dentistry, children

## Abstract

**Background:** Understanding predictors of pain associated with paediatric dental procedures could play an important role in preventing loss of cooperation, which often leads to the procedure having to be performed under general anaesthesia. **Aim:** We aimed to identify predictors of intra-operative and post-operative pain associated with routine dental procedures in children. **Materials and Methods:** A systematic review of observational studies was performed using electronic searches on MEDLINE, EMBASE, PsycINFO, Global Health via OVID, PubMed, Scopus, and SciELO. The NIH Quality Assessment Tool for Observational Cohort and Cross-Sectional Studies was used to evaluate the quality of the included studies, which were meta-analysed to estimate the impact of dental procedures and anxiety on children’s pain perception. A meta-regression analysis was also performed to determine the relative effect of predictors on children’s pain perception measured as mean differences on a visual analogue scale (VAS). **Results:** The search identified 532 articles; 53 were retrieved for full-text screening; 6 studies were included in the review; and 4 were eligible for the meta-analysis. The meta-analysis showed the types of procedures that predicted intra-operative pain, with dental extractions being the most painful (Mean VAS Difference [MD] 46.51 mm, 95% confidence interval [CI] 40.40 to 52.62 mm). The meta-regression showed that pain scores for dental extractions were significantly higher than polishing (the least painful procedure (reference category)) by VAS MD = 23.80 mm (95% CI 5.13–42.46 mm, *p*-value = 0.012). It also showed that highly anxious children reported significantly higher pain scores during dental procedures by a 12.31 mm MD VAS score (95% CI 5.23–19.40 mm, *p*-value = 0.001) compared to those with low anxiety levels. **Conclusions:** This systematic review demonstrates that the strongest predictors of intra-operative pain associated with paediatric dental procedures are dental extractions followed by drilling. Children with high anxiety also reported more pain for similar procedures. Tailoring interventions to reduce pain associated with paediatric dental procedures should be a priority for future research, as reducing pain can impact compliance and could reduce the need for general anaesthesia in dental treatment.

## 1. Introduction

Dental treatment can cause pain during and after the completion of the procedure, which may prevent some children from receiving optimal dental care. Children might describe dental procedures as painful and unpleasant experiences [1]. The effective management of pain is imperative, particularly in children and adolescents, because painful and unpleasant dental experiences may hinder children from seeking further treatment [2]. Other consequences of painful and unpleasant dental experiences include anxiety, fear, a lack of cooperation, delay or avoidance of seeking further dental care, and the need for general anaesthesia during dental treatment [1,2].

Pain due to dental caries is considered one of the most common reasons for hospital admissions among children who need to be treated under general anaesthesia (GA) in the UK [3]. Although there are some benefits to having dental treatment under GA, there are associated risks of morbidity and mortality. While there is a minimal risk of death from GA for dental treatment (approximately 1 in 250,000), morbidities such as nausea, vomiting, post-operative pain, and injuries to adjacent teeth and structures are significantly more common [3,4]. It has been found that 40 to 90% of children having dental general anaesthesia experienced pain, headache, nausea, vomiting, sore throat, sleepiness, and bleeding [5].

Dental general anaesthesia (DGA) is a significant emotional event that children may experience [6]. It has been found that dental treatment under GA can be a traumatic experience for children due to the stressful procedures associated with GA, such as induction, the relative loss of control, sequencing of events, or the unfamiliar environment and personnel [6,7]. General anaesthesia for dental treatment has been thought of as a contributing factor to dental fear and anxiety (DFA) both in the short term and long term [6,7,8,9,10,11,12].

Dental treatment under GA is considered expensive and resource-intensive, which places a considerable financial burden on the National Health Service (NHS) in the UK and other countries [3,13]. For example, it was reported that about 43,700 children aged ≤16 years were admitted to hospitals in England in 2015–2016 for the extraction of multiple teeth under GA, which cost GDP 30 million [3]. In 2018–2019, 44,685 surgical procedures were carried out in English hospitals to remove more than one tooth in children aged 18 years and under, which cost GDP 41.5 million [13].

Long waiting times for paediatric dental procedures under GA are another significant issue [3,13]. It has been found that children may wait over a year to be treated under GA [14]. These children may experience multiple episodes of pain, distress, and infection during this time [13,15]. Several visits to primary dental care may be necessary for placing a number of temporary dressings or prescribing courses of antibiotics to relieve pain and infection during this period [13,15].

Because of the risks, costs, and availability, GA for dental care should only be used when other options have failed or are not appropriate. Understanding and reducing intra-operative and post-operative pain associated with dental procedures can improve the dental experience and may avoid the need for GA. Therefore, the aim of this systematic review was to investigate predictors of intra-operative and post-operative pain associated with routine paediatric dental procedures.

## 2. Material and Methods

### 2.1. Protocol Registration and Review Reporting

This systematic review and meta-analysis were undertaken following the Preferred Reporting Items for Systematic Reviews and Meta-Analyses (PRISMA) statement [16,17]. The protocol was registered and published in the International Prospective Register of Systematic Reviews (PROSPERO; registration number: CRD42020177746).

### 2.2. Search Strategy

This review used electronic searches with detailed search strategies developed for each database searched to identify eligible studies. The search strategies were formulated by the author (M.A.A.) under the supervision of a specialist librarian at the University of Leeds (Supplementary File). A combination of controlled vocabulary and free-text terms was considered for the search strategy to identify eligible studies with no restrictions regarding language or the date of publication. The following electronic databases were searched up to 18 December 2023: MEDLINE via OVID, EMBASE via OVID and PsycINFO via OVID, Global Health via OVID, PubMed, Scopus and SciELO (Web of Science). The included studies’ references were also screened to identify additional eligible studies. Hand-searching in related dental journals was carried out when electronic copies were not available.

### 2.3. Inclusion Criteria

All observational studies, such as cross-sectional and cohort studies that investigated predictors of pain in routine paediatric dental procedures, were included. Routine paediatric dental procedures such as a diagnostic examination, probing, scaling, polishing, radiograph, local anaesthesia, drilling, restoration, pulpotomy/pulpectomy, and extraction were included. Other predictors such as age, gender, infection, anxiety, previous dental and medical experience, and dentists’ knowledge and attitudes to pain were also included. Studies involving children and adolescents aged up to 19 years of age, regardless of medical and behavioural problems, were included.

### 2.4. Exclusion Criteria

Studies involving dental treatment under sedation (including nitrous oxide) or general anaesthesia were excluded.

### 2.5. Outcome

The outcomes that were considered for this review were intra-operative and post-operative pain measured using a visual analogue scale (VAS) or other validated scales such as the Faces Pain Scale.

### 2.6. Data Selection

All references were exported into EndNote version X9 (Clarivate, Philadelphia, PA, USA); then, the studies were imported into the Covidence systematic review software, where duplicated records were identified and removed. Covidence is a custom-built data system designed to assist reviewers in using a structured data collection form for online form building, data entry, data sharing, and efficient data management [18,19]. The titles and abstracts of relevant articles were independently assessed by the two review authors (M.A.A. and A.J.). Discussion and consensus were considered to resolve any disagreement; a third reviewer (VRA) was consulted when consensus was not achieved.

### 2.7. Data Extraction

Any study that met the eligibility criteria regardless of the study quality was included. The study authors were contacted for more details when there were missing data or inconsistent reporting. The required information was extracted in duplicate by the two reviewers (M.A.A. and A.J.) using the Covidence systematic review software. The following study characteristics were obtained:

○ Name of the first author.
○ Journal of publication.
○ Year of publication.
○ Country.
○ Sitting.
○ Study design.
○ Population and participant characteristics.
○ Sample size.
○ Predictors.
○ The type of outcome.
○ Methods of measurement.

### 2.8. Quality Assessment

The quality assessment of the included studies was undertaken independently by the same reviewers (M.A.A. and A.J.). The NIH Quality Assessment Tool for Observational Cohort and Cross-Sectional Studies was considered because the included studies in this systematic review used observational (cohort and cross-sectional) designs as stated in the protocol [20]. This tool consists of 14 questions, which are designed to assist reviewers in focusing on concepts that are important to a study’s internal validity, such as the sample characteristics, recruitment process, and the level of in-depth reported information on the exposure and outcome measures [8]. Following the assessment, the studies were then assessed as ‘Good’, ‘Fair’, or ‘Poor’. A numeric score was created to facilitate the rating of overall quality for each study based on the number of ‘Yes’ responses to the assessment questions. The grading was then decided based on the total score: 0–4 (Poor), 5–9 (Fair) and 10–14 (Good). None were excluded based on their quality rating. Discussion and consensus were considered to resolve any disagreement, and a third reviewer (VRA) was consulted when consensus was not achieved.

### 2.9. Data Analysis

All extracted data were exported and managed in an Excel file (Microsoft Inc., Redmond, WA, USA). The data extraction form was then modified to facilitate the process of data analysis.

Meta-analysis was carried out if there were sufficient studies reporting the same outcomes to give more power to combine the results of these studies. The mean differences (MDs) and 95% confidence intervals (CIs) were considered for continuous outcomes that were measured with the same scale to estimate the impact of predictors on children’s pain perception. Data collection for the meta-analysis included the following information:

○ Name of the first author.
○ Dental procedures included.
○ Anxiety levels (high/low).
○ Pain outcomes (mean, standard of deviation (SD), number of participants (N), and Standard of error (SE)).

Forest plots were generated using Stata 16 statistical software (StataCorp LLC, College Station, TX, USA) and a random-effects model. The random-effects model was considered because of the large heterogeneity that was expected to be found across studies and was later corroborated by the analysis. Meta-regression analysis using a random-effects model was also performed to determine the relative effect of the predictors on children’s pain perception compared to a reference category, which was chosen as the least painful procedure for dental procedures and low anxiety levels for anxiety.

The clinical heterogeneity of the included studies was accounted for by the inclusion criteria for studies, participants, components of the predictors, and outcome measures. I^2^ statistics were used for statistical heterogeneity; I^2^ statistics with values of 50% or greater represented substantial heterogeneity. A *p*-value ≤ 0.05 was considered statistically significant.

The clinically important difference in pain intensity was determined by a change of 10 for the 100 mm pain VAS [21,22]. Therefore, if the MD VAS score was 10 mm or more, it was considered clinically significant.

## 3. Results

### 3.1. Study Selection

**Figure 1:**
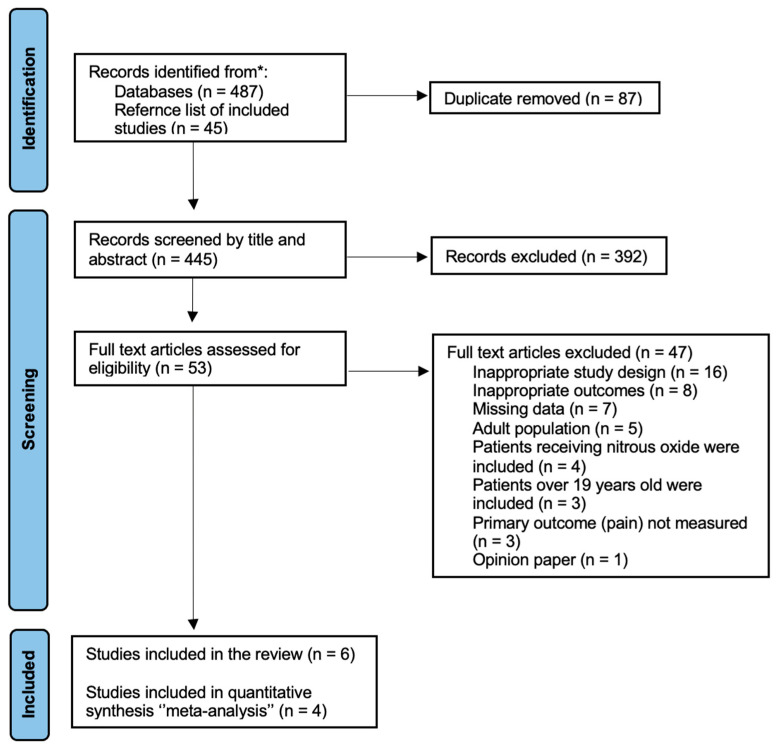
PRISMA flowchart of the study selection process. * MEDLINE via OVID, EMBASE via OVID and PsycINFO via OVID, Global Health via OVID, PubMed, Scopus and SciELO (Web of Science).

A total of 532 studies were identified in the electronic and manual searches, with 445 remaining after excluding duplicates. Following the title and abstract screening, 53 articles were selected for a full-text review and examined against the eligibility criteria in detail. Forty-seven studies were excluded on the basis of an inappropriate study design (n = 16), the outcome of interest not being recorded (n = 11), adult participants (n = 8), missing data (n = 7), participants receiving treatment under nitrous oxide inhalation sedation (n = 4), and one study was an opinion paper. Consequently, six studies were identified and included in the review, and four studies were eligible for the meta-analysis (Figure 1).

### 3.2. Study Characteristics

The main characteristics of the included papers are summarised in Table 1.

A cross-sectional study design was adopted in three studies [23,24,25], whereas a cohort study design was used in the other studies [1,26,27]. Participant ages ranged from three to nineteen years. The studies included different sample sizes ranging from 36 to 2363 children. Of the six eligible studies, two [1,26] were conducted in Sweden, one [27] in Canada, one [24] in India, one [23] in Brazil, and one in the Netherlands [25].

The eligible studies Involved dental procedures, including check-ups, diagnostic examinations, radiographs, probing, scaling, polishing, local anaesthesia, drilling, restorations, and/or extractions.

All studies reported on intra-operative pain, and none of the studies included data on post-operative pain. Three studies [25,26,27] measured anxiety levels before the dental procedure, and one study [25] compared intra-operative pain between two different age groups. Pain intensity was measured using a visual analogue scale [28] in two studies [1,26] and a modified version of the visual analogue scale in one study [25]. Two studies [23,27] used a revised version of the Faces Pain Scale (FPS-R) [29], and one study [24] used the face, legs, activity, cry, and consolability (FLACC) scale [29]. Anxiety was measured by the parent’s version of the dental subscale of the children’s fear survey schedule (CFSS-DS) in one study [25], the Dental Anxiety Scale in one study [26], and the trait anxiety portion of the Spielberger State-Trait Anxiety Inventory for Children in one study [27].

### 3.3. Quality Assessment of the Studies

The findings regarding the quality assessment for the included studies are summarised in Table 2. All studies were considered as being of a good quality level overall except for one study [27], which was rated as a fair quality level. The criteria “Participation rate > 50%’’ and “Loss to follow-up after baseline 20% or less” could not be determined in one study [27]. Only two studies [25,27] did not justify their sample size. All studies did not measure exposures prior to outcomes because the exposures and outcomes were measured during the same timeframe. The answer for the criterion “sufficient timeframe” was no for the cross-sectional studies, as this study design assessed the exposures and outcomes at the same time [23,24,25]. Two studies [23,24] did not assess exposure more than once over time. The outcome assessors were not blinded in three studies [1,24,25], while it could not be determined whether the outcome assessors were blinded or not in the remaining studies [23,26,27].

### 3.4. Meta-Analysis of Intra-Operative Pain Outcome

**Figure 2:**
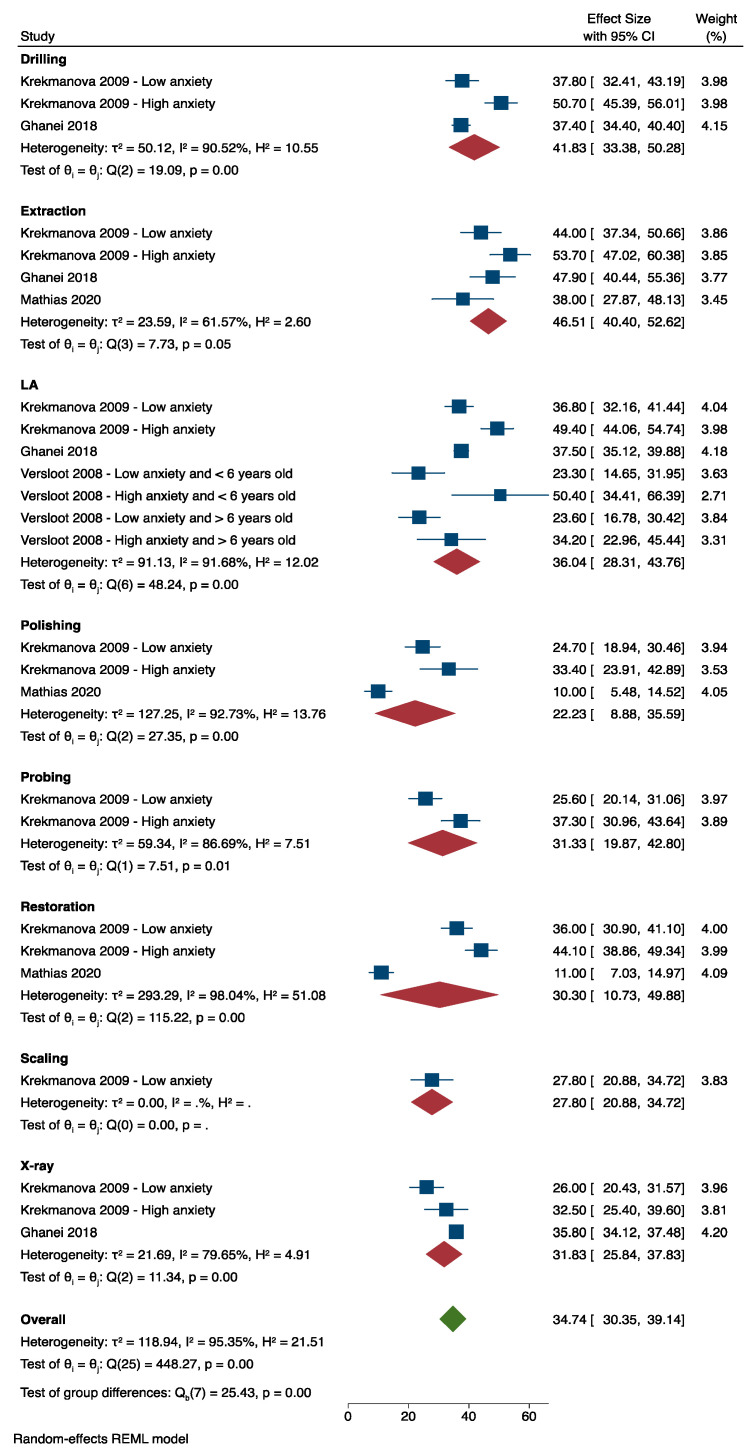
Random-effects meta-analysis evaluation of pain associated with dental procedures.

Figure 2 shows the main results of the meta-analysis of intra-operative pain outcomes. Four studies [1,23,25,26] were included in the meta-analysis. The subgroup analysis of dental procedures showed that the most painful procedure was extraction (MD VAS 46.51 mm, 95% CI 40.40 mm to 52.62 mm) followed by drilling (MD VAS 41.83 mm, 95% CI 33.38 mm to 50.28 mm), and local anaesthesia (MD VAS 36.04 mm, 95% CI 28.31 mm to 43.76 mm). The heterogeneity was generally high for each subgroup; thus, the random-effects model was the best approach to pool the data of the included studies. Not all studies from Table 1 could be included in the meta-analysis as they used different pain scales to measure outcomes, e.g., categorical versus continuous.

### 3.5. Meta-Regression of Intra-Operative Pain Outcome

The results for the meta-regression of dental procedures are shown in Table 3.

### 3.6. Dental Procedures

Polishing was considered as a reference score for the meta-regression of dental procedures because it was the least painful procedure; the MD VAS pain score for polishing was 22.28 mm, 95% CI 8.24 to 36.31 mm. The results demonstrated that the MD VAS pain score for dental extraction was higher than polishing by 23.80 mm, with a 95% CI of 5.13 mm to 42.46 mm, which was statistically and clinically significant (*p*-value = 0.012 and the MD VAS score was >10 mm). The mean pain score for drilling was also found to be higher than polishing by 19.64 mm, with a 95% CI of 0.001 mm to 39.28 mm, which was statistically and clinically significant (*p*-value = 0.05 and the MD VAS score was >10 mm). Although the mean VAS pain score for LA was not statistically significant (*p*-value = 0.108), it was reported to be more painful than polishing by 13.84 mm, with a 95% CI of −3.03 to 30.72 mm, i.e., a clinically significant finding as the MD VAS score was >10 mm.

### 3.7. Anxiety Levels

The meta-regression of anxiety levels used low anxiety levels as a reference score because they were reported with a lower mean pain score of 30.73, with a 95% CI of 25.99 to 35.46. The results showed that children with high anxiety levels reported significantly higher mean pain scores by 12.31 mm, with a 95% CI of 5.23 to 19.40 mm and a *p*-value of 0.001 compared to those with low anxiety levels (Table 4).

## 4. Discussion

The aim of this systematic review was to identify predictors of intra-operative and post-operative pain associated with routine dental treatment in children. Data were assembled from six studies comprising 3213 children, of which four were included in the meta-analysis. The findings revealed that dental procedures were strong predictors of intra-operative pain and that the strongest predictor was extraction, followed by drilling when the pain was assessed intra-operatively. High anxiety levels were also found to be a predictor of intra-operative pain associated with paediatric dental treatment.

These predictors are important for dental practitioners to consider when they provide dental treatment for children. Dental practitioners should carefully listen to and be aware of the implications of the responses of their paediatric patients in order to provide acceptable and effective anaesthesia so that procedures can be completed with as little pain as possible. They should also assess their young patients pre-operatively for dental anxiety in order to use appropriate anxiety management techniques. It is important for general dental practitioners and paediatric dentists to identify children with dental anxiety from an early stage at a new patient appointment. A systematic review carried out by Porritt et al. in 2013 assessing children’s dental anxiety recommended using a dental anxiety measure that involves different specific questions related to dental procedures suitable for a wide range of ages, and it can be completed in the waiting room [30]. They also provided different useful dental anxiety measures that have these desirable properties and can be used in primary dental care, such as the Modified Child Dental Anxiety Scale (MCDAS), Smiley Faces Programme (SFP), Dental Fear Survey (DFS) and Facial Image Scale (FIS).

It is well recorded in the literature that exposing children to a painful dental procedure may have a variety of adverse consequences, such as anxiety, fear, lack of cooperation, delay in seeking dental care, or the need for sedation or general anaesthesia for dental treatment. Fear of pain has been thought to be a source of anxiety, which could make children postpone seeking further dental treatment [31]. It has also been shown that patients who have experienced painful dental treatment may face some difficulties in their treatment and may avoid future dental care [32]. Similarly, it has been reported that repeated painful experiences during dental treatment are one of the leading reasons for dental practitioners to consider GA for delivering dental care to some children [33]. Despite some benefits of having dental treatment under GA, there are associated risks and complications. Therefore, a good understanding of the predictors of pain associated with routine dental procedures can play an important role in reducing the number of children requiring GA for their dental treatment by using appropriate pharmacological and non-pharmacological interventions to target painful procedures to help relieve pain and anxiety.

The main strength of the present systematic review is that it is the first comprehensive systematic review to investigate the predictors of pain associated with routine paediatric dental procedures, reporting the main predictors of intra-operative pain. Additionally, the quality standards according to PRISMA were employed in this review [16,17], and a broad search strategy of several databases without language and date restrictions was considered. This allowed the reviewers to identify and include many potentially eligible studies, therefore minimising the risk of selection bias [34]. Furthermore, the reviewers independently assessed studies for eligibility, extracted data, and evaluated the quality of the included studies to minimise selection and information bias and error to improve the reliability and validity of this review. The primary outcome of this review was intra-operative and post-operative pain. The included studies measured intra-operative pain using different pain scales. The pain scales consisted of the VAS, the modified version of VAS and the VAS (FPS-R), and the FLACC. The self-report measure of pain has been considered the gold standard for assessing pain in children [28]. The FLACC scale is a pain assessment scale used when a self-report of pain is not applicable, and it assesses pain through the observation of five categories, including the face, legs, activity, cry, and consolability [29]. The measurement of pain may be influenced by the child’s anxiety [25]. The present review included two large, good-quality studies [25,26] with sufficient sample sizes that measured anxiety before dental treatment using the DAS and the parent’s version of the CFSS-DS, respectively.

Some limitations are noted in this review. None of the included studies measured post-operative pain, as their main interest, was to assess intra-operative pain associated with dental procedures, and the participants were not followed up for any presence or absence of post-operative pain. Therefore, it is important for future research to consider measuring post-operative pain associated with routine dental treatment in children, as this pain may impact future dental care. Although the overall quality assessment was good for the majority of the included studies in this review, flaws were identified in the methodology of included studies. The participation rate could not be determined in one study as to whether it was more or less than 50% of eligible children who participated. If the participation rate was less than 50%, this raises concern that the study population does not adequately represent the target population [20]. Also, the sample size was not justified in the two studies. However, the lack of sample size justification was not considered a fatal flaw as these studies were exploratory [20], and the samples of a number of studies were combined in this review to increase the level of statistical power. The blinding of outcome assessors was not achieved in half of the studies, and it could not be determined in the remaining studies. This could introduce some bias as the assessor may influence the participant and the subsequent results [35]. However, the outcome of most of the included studies in this review was measured using the self-report measure of pain, which reduces the chance of detection bias. Whilst only six studies were included, which could affect generalizability, sample sizes were large, and the studies represented several countries from both high and low-middle-income settings.

## 5. Conclusions

This systematic review demonstrates that dental extraction and drilling are the most common predictors of intra-operative pain associated with routine dental treatment in children. It also shows that children with high anxiety levels reported more intra-operative pain for similar procedures. A good understanding of the predictors of pain associated with routine dental procedures could play an important role in providing appropriate pharmacological and behavioural support to help children cope better with dental care. This, in turn. could reduce the number of children requiring general anaesthesia for dental treatment by using appropriate pharmacological and non-pharmacological interventions to target these predictors and reduce the pain and anxiety associated with dental procedures. Further research is needed to understand predictors of post-operative pain as none of the identified studies measured post-operative pain.

### Why This Paper Is Important to Paediatric Dentists?

- Having a good understanding of the predictors of pain associated with routine dental treatment in children can help general dental practitioners and paediatric dentists choose and provide appropriate pharmacological and/or non-pharmacological interventions to help relieve pain and anxiety and allow children to cope better with dental care.
- These interventions should be a priority for future research and implementation as they could help children to cooperate during dental procedures, thereby preventing the need for general anaesthesia, which is costly, resource intensive, and has associated risks of morbidity and mortality.

## Figures and Tables

**Table 1:** The characteristics of included studies.

Included Studies	Country	Setting	Design	Sample Size	Age	Intervention	Outcome Variable	Outcome Measure
Krekmanova 2009 [23]	Sweden	Three Public Dental Service clinics in the city of Goteborg	Retrospective cohort study	368	8–19 years old	Extraction, drilling, LA, restoration, scaling, probing, X-ray, and polishing	Pain and Anxiety	0–100 visual analogue scale for pain and Dental Anxiety Scale for anxiety
Rocha 2009 [24]	Canada	Six dental practices serving families from both urban and rural settings	Prospective cohort study	36	5–12 years old	Dental procedures (e.g., polishings, check-ups, diagnostic examinations, fillings, and extractions)	Pain and Anxiety	0–10 VAS, Faces Pain Scale-Revised (FPS-R) and the trait anxiety portion of the Spielberger State-Trait Anxiety Inventory for Children
Ghanei 2018 [1]	Sweden	Seven Public Dental Service clinics in RVG and five in ROC	Prospective cohort study	2363	3–19 years old	LA, extraction, drilling, and X-ray	Pain	0–10 visual analogue scale
Mathias 2020 [25]	Brazil	The Paediatric Dentistry Clinic of the School of Dentistry at the Federal University of Pelotas	Cross-sectional study	192	6–13 years old	Polishing, restoration, and extraction	Pain	0–10 VAS, Faces Pain Scale-Revised (FPS-R)
Pala 2016 [26]	India	Narayana Dental College and Hospital	Cross-sectional study	107	4–13 years old	LA and extraction	Pain	0–10 Face, legs, activity, cry, consolability scale
Versloot 2008 [27]	Netherlands	A special dental care clinic	Cross-sectional study	147	4–11 years old	LA	Pain and Anxiety	0–10 Modified version of the VAS for pain and the parent’s version of the dental subscale of the children’s fear survey schedule for anxiety

**Table 2:** Quality assessment summary.

Included Studies	Clear Aim	Sample Defined	Participation Rate > 50%	Inclusion and Exclusion Criteria	Sample Size Justification	Exposure Measured Prior to Outcome	Sufficient Time Frame	Levels of Exposure	Exposure Measures	Exposure Assessed More Than Once Over Time	Outcome Measures	Assessors Blinding	Loss to Follow-Up after Baseline 20% or Less	Adjusted for Confounders	Total Score *	Quality Rating *
Krekmanova 2009 [26]	Yes	Yes	Yes	Yes	Yes	No	Yes	Yes	Yes	Yes	Yes	CD *	Yes	Yes	12/14	Good
Rocha 2009 [27]	Yes	Yes	CD *	Yes	No	No	Yes	Yes	Yes	Yes	Yes	CD *	CD *	Yes	9/14	Fair
Ghanei 2018 [1]	Yes	Yes	Yes	Yes	Yes	No	Yes	Yes	Yes	Yes	Yes	No	Yes	Yes	12/14	Good
Mathias 2020 [23]	Yes	Yes	Yes	Yes	Yes	No	No	Yes	Yes	No	Yes	CD *	Yes	Yes	10/14	Good
Pala 2016 [24]	Yes	Yes	Yes	Yes	Yes	No	No	Yes	Yes	No	Yes	No	Yes	Yes	10/14	Good
Versloot 2008 [25]	Yes	Yes	Yes	Yes	No	No	No	Yes	Yes	Yes	Yes	No	Yes	Yes	10/14	Good

* CD, cannot determine. * Total score: number of yeses. * Quality rating: 0–4 (poor), 5–9 (fair) and 10–14 (good).

**Table 3:** Random-effects meta-regression evaluation of pain associated with dental procedures.

Dental Procedures (N = 26)	Mean Pain Score Difference (95% CI)	*p*-Value
Drilling [1,26]	19.64 (0.001–39.28)	0.05
Extraction [1,23,26]	23.80 (5.13–42.46)	0.012
LA [1,25,26]	13.84 (−3.03–30.72)	0.108
Probing [26]	9.12 (−12.94–31.18)	0.418
Restoration [23,26]	7.96 (−11.69–27.61)	0.427
Scaling [26]	5.52 (−22.57–33.62)	0.700
Radiograph [1,26]	9.22 (−10.47–28.91)	0.359
Polishing (the reference score) [23,26]		

**Table 4:** Random-effects meta-regression evaluation of pain associated with anxiety level.

Anxiety Level	Mean Pain Score (95% CI)	*p*-Value
High anxiety	12.31 (5.23–19.40)	0.001
Low anxiety (the reference score)		

## Supplementary Materials

The search strategy information can be downloaded at: https://www.mdpi.com/article/10.3390/dj12010004/s1.
