# Supplementary material for: Predictors of Intra-Operative and Post-Operative Pain Associated with Routine Dental Procedures in Children: A Systematic Review and Meta-Analysis
Source: Dent J (Basel). 2023 Dec 25;12(1):4. doi: 10.3390/dj12010004 (PMC10814801; doi:10.3390/dj12010004)
Supplement: Supplementary file 1 [file dentistry-12-00004-s001.zip › dentistry-2717646-supplementary.pdf]

Supplementary File:

Search Strategy:

MEDLINE via OVID
1. exp Dentistry/
2. (dental* or dentist*).ti,ab.
3. (oral adj5 surg*).ti,ab.
4. (orthodontic* or pulpotom* or pulpect* or endodont* or "pulp cap*").mp.
5. ((dental or tooth or teeth) adj5 (fill* or restor* or extract* or remov* or "cavity prep*" or caries or carious or decay*)).mp.
6. (root canal and (therap* or treat*)).mp.
7. child/
8. Infant/
9. adolescent/
10. Pediatrics/
11. Dental care for children/
12. (child* or adolescent* or kid or kids or youth* or youngster* or minor or minors or teen* or preteen* or pre-teen* or juvenile* or "young adult*" or "young person" or "young people" or p?ediatric* or student* or pupil or pupils or boy or boys or girl or girls or under 18* or under eighteen* or underage).ti,ab,kw.
13. Pain, Postoperative/
14. (postoperative adj4 pain*).ti,ab,kw.
15. (post-operative adj4 pain*).ti,ab,kw.
16. post-operative-pain*.ti,ab,kw.
17. (post* adj4 pain*).ti,ab,kw.
18. (postoperative adj4 analgesi*).ti,ab,kw.
19. (post-operative adj4 analgesi*).ti,ab,kw.
20. post-operative analgesi*.ti,ab,kw.
21. (post-surgical adj4 pain*).ti,ab,kw.
22. (post surgical adj4 pain*).ti,ab,kw.
23. (pain* adj4 after surg*).ti,ab,kw.
24. (pain* adj4 after operat*).ti,ab,kw.
25. (pain* adj4 follow* operat*).ti,ab,kw.
26. (pain* adj4 follow* surg*).ti,ab,kw.
27. (pain adj1 operati*).ti,ab,kw.
28. (intra adj procedur* adj pain).ti,ab,kw.
29. (intraoperative adj4 pain*).ti,ab,kw.
30. (intra-operative adj4 pain*).ti,ab,kw.
31. intra-operative-pain*.ti,ab,kw.
32. (intraoperative adj4 analgesi*).ti,ab,kw.
33. (intra-operative adj4 analgesi*).ti,ab,kw.
34. intra-operative analgesi*.ti,ab,kw.
35. (intra-surgical adj4 pain*).ti,ab,kw.
36. (intrasurgical adj4 pain*).ti,ab,kw.
37. (pain* adj4 after surg*).ti,ab,kw.
38. (pain* adj4 after operat*).ti,ab,kw.
39. (pain* adj4 follow* operat*).ti,ab,kw.
40. (pain* adj4 follow* surg*).ti,ab,kw.
41. (surg* adj1 pain).ti,ab,kw.
42. (("post surg*" or post-surg*) and (pain* or discomfort)).ti,ab,kw.
43. (analgesi* adj4 surg*).ti,ab,kw.
44. (analgesi* adj4 operat*).ti,ab,kw.

45. (sore* or hurt* or ache* or aching or discomfort* or uncomfort* or tender* or throb*).mp. [mp=title, abstract, original title, name of substance word, subject heading word, floating sub-heading word, keyword heading word, organism supplementary concept word, protocol supplementary concept word, rare disease supplementary concept word, unique identifier, synonyms]
46. predictor*.tw,kw.
47. Protective factors/
48. risk assessment/
49. risk factors/
50. (Risk adj factor*).tw,kw.
51. (risk adj assessment*).tw,kw.
52. (protective adj factor*).tw,kw.
53. Prevalence/
54. Prevalence.tw,kw.
55. Incidence/
56. Incidence.tw,kw.
57. Prognosis/
58. Prognos*.tw,kw.
59. correlati*.tw,kw.
60. An?esthetics, Local/
61. An?esthesia, Local/
62. (local adj5 (anesthetic* or anaesthetic* or anesthesia or anaesthesia)).mp.
63. Lidocaine/
64. (lidocaine or lignocaine or xylocaine).mp.
65. Carticaine/
66. (carticain* or articain*).mp.
67. Prilocaine/
68. (prilocain* or citanest* or propitocain* or xylonest).mp.
69. Bupivacaine/
70. (bupivacain* or buvacaina or carbostesin or dolanaest or marcain* or sensorcain* or svedocain*).mp.
71. 1 or 2 or 3 or 4 or 5 or 6
72. 7 or 8 or 9 or 10 or 11 or 12
73. 13 or 14 or 15 or 16 or 17 or 18 or 19 or 20 or 21 or 22 or 23 or 24 or 25 or 26 or 27 or 28 or 29 or 30 or 31 or 32 or 33 or 34 or 35 or 36 or 37 or 38 or 39 or 40 or 41 or 42 or 43 or 44 or 45
74. 46 or 47 or 48 or 49 or 50 or 51 or 52 or 53 or 54 or 55 or 56 or 57 or 58 or 59
75. 60 or 61 or 62 or 63 or 64 or 65 or 66 or 67 or 68 or 69 or 70
76. 71 and 72 and 73 and 74 and 75

EMBASE via OVID

1. exp Dentistry/
2. (dental* or dentist*).ti,ab.
3. (oral adj5 surg*).ti,ab.
4. (orthodontic* or pulpotom* or pulpect* or endodont* or "pulp cap*").mp.
5. ((dental or tooth or teeth) adj5 (fill* or restor* or extract* or remov* or "cavity prep*" or caries or carious or decay*)).mp.
6. (root canal and (therap* or treat*)).mp.
7. child/
8. Infant/
9. adolescent/
10. Pediatrics/
11. Dental care for children/

12. (child* or adolescent* or kid or kids or youth* or youngster* or minor or minors or teen* or preteen* or pre-teen* or juvenile* or "young adult*" or "young person" or "young people" or p?ediatric* or student* or pupil or pupils or boy or boys or girl or girls or under 18* or under eighteen* or underage).ti,ab,kw.
13. Pain, Postoperative/
14. (postoperative adj4 pain*).ti,ab,kw.
15. (post-operative adj4 pain*).ti,ab,kw.
16. post-operative-pain*.ti,ab,kw.
17. (post* adj4 pain*).ti,ab,kw.
18. (postoperative adj4 analgesi*).ti,ab,kw.
19. (post-operative adj4 analgesi*).ti,ab,kw.
20. post-operative analgesi*.ti,ab,kw.
21. (post-surgical adj4 pain*).ti,ab,kw.
22. (post surgical adj4 pain*).ti,ab,kw.
23. (pain* adj4 after surg*).ti,ab,kw.
24. (pain* adj4 after operat*).ti,ab,kw.
25. (pain* adj4 follow* operat*).ti,ab,kw.
26. (pain* adj4 follow* surg*).ti,ab,kw.
27. (pain adj1 operati*).ti,ab,kw.
28. (intra adj procedur* adj pain).ti,ab,kw.
29. (intraoperative adj4 pain*).ti,ab,kw.
30. (intra-operative adj4 pain*).ti,ab,kw.
31. intra-operative-pain*.ti,ab,kw.
32. (intraoperative adj4 analgesi*).ti,ab,kw.
33. (intra-operative adj4 analgesi*).ti,ab,kw.
34. intra-operative analgesi*.ti,ab,kw.
35. (intra-surgical adj4 pain*).ti,ab,kw.
36. (intrasurgical adj4 pain*).ti,ab,kw.
37. (pain* adj4 after surg*).ti,ab,kw.
38. (pain* adj4 after operat*).ti,ab,kw.
39. (pain* adj4 follow* operat*).ti,ab,kw.
40. (pain* adj4 follow* surg*).ti,ab,kw.
41. (surg* adj1 pain).ti,ab,kw.
42. (("post surg*" or post-surg*) and (pain* or discomfort)).ti,ab,kw.
43. (analgesi* adj4 surg*).ti,ab,kw.
44. (analgesi* adj4 operat*).ti,ab,kw.
45. (sore* or hurt* or ache* or aching or discomfort* or uncomfort* or tender* or throb*).mp. [mp=title, abstract, heading word, drug trade name, original title, device manufacturer, drug manufacturer, device trade name, keyword, floating subheading word, candidate term word]
46. predictor*.tw,kw.
47. Protective factors/
48. risk assessment/
49. risk factors/
50. (Risk adj factor*).tw,kw.
51. (risk adj assessment*).tw,kw.
52. (protective adj factor*).tw,kw.
53. Prevalence/
54. Prevalence.tw,kw.
55. Incidence/
56. Incidence.tw,kw.
57. Prognosis/
58. Prognos*.tw,kw.
59. correlati*.tw,kw.
60. An?esthetics, Local/
61. An?esthesia, Local/

62. (local adj5 (anesthetic* or anaesthetic* or anesthesia or anaesthesia)).mp.
63. Lidocaine/
64. (lidocaine or lignocaine or xylocaine).mp.
65. Carticaine/
66. (carticain* or articain*).mp.
67. Prilocaine/
68. (prilocain* or citanest* or propitocain* or xylonest).mp.
69. Bupivacaine/
70. (bupivacain* or buvacaina or carbostesin or dolanaest or marcain* or sensorcain* or svedocain*).mp.
71. 1 or 2 or 3 or 4 or 5 or 6
72. 7 or 8 or 9 or 10 or 11 or 12
73. 13 or 14 or 15 or 16 or 17 or 18 or 19 or 20 or 21 or 22 or 23 or 24 or 25 or 26 or 27 or 28 or 29 or 30 or 31 or 32 or 33 or 34 or 35 or 36 or 37 or 38 or 39 or 40 or 41 or 42 or 43 or 44 or 45
74. 46 or 47 or 48 or 49 or 50 or 51 or 52 or 53 or 54 or 55 or 56 or 57 or 58 or 59
75. 60 or 61 or 62 or 63 or 64 or 65 or 66 or 67 or 68 or 69 or 70
76. 71 and 72 and 73 and 74 and 75

PsycINFO via OVID

1. exp Dentistry/
2. (dental* or dentist*).ti,ab.
3. (oral adj5 surg*).ti,ab.
4. (orthodontic* or pulpotom* or pulpect* or endodont* or "pulp cap*").mp.
5. ((dental or tooth or teeth) adj5 (fill* or restor* or extract* or remov* or "cavity prep*" or caries or carious or decay*)).mp.
6. (root canal and (therap* or treat*)).mp.
7. child/
8. Infant/
9. adolescent/
10. Pediatrics/
11. Dental care for children/
12. (child* or adolescent* or kid or kids or youth* or youngster* or minor or minors or teen* or preteen* or pre-teen* or juvenile* or "young adult*" or "young person" or "young people" or p?ediatric* or student* or pupil or pupils or boy or boys or girl or girls or under 18* or under eighteen* or underage).ti,ab,kw.
13. Pain, Postoperative/
14. (postoperative adj4 pain*).ti,ab,kw.
15. (post-operative adj4 pain*).ti,ab,kw.
16. post-operative-pain*.ti,ab,kw.
17. (post* adj4 pain*).ti,ab,kw.
18. (postoperative adj4 analgesi*).ti,ab,kw.
19. (post-operative adj4 analgesi*).ti,ab,kw.
20. post-operative analgesi*.ti,ab,kw.
21. (post-surgical adj4 pain*).ti,ab,kw.
22. (post surgical adj4 pain*).ti,ab,kw.
23. (pain* adj4 after surg*).ti,ab,kw.
24. (pain* adj4 after operat*).ti,ab,kw.
25. (pain* adj4 follow* operat*).ti,ab,kw.
26. (pain* adj4 follow* surg*).ti,ab,kw.
27. (pain adj1 operati*).ti,ab,kw.
28. (intra adj procedur* adj pain).ti,ab,kw.
29. (intraoperative adj4 pain*).ti,ab,kw.
30. (intra-operative adj4 pain*).ti,ab,kw.
31. intra-operative-pain*.ti,ab,kw.

32. (intraoperative adj4 analgesi*).ti,ab,kw.
33. (intra-operative adj4 analgesi*).ti,ab,kw.
34. intra-operative analgesi*.ti,ab,kw.
35. (intra-surgical adj4 pain*).ti,ab,kw.
36. (intrasurgical adj4 pain*).ti,ab,kw.
37. (pain* adj4 after surg*).ti,ab,kw.
38. (pain* adj4 after operat*).ti,ab,kw.
39. (pain* adj4 follow* operat*).ti,ab,kw.
40. (pain* adj4 follow* surg*).ti,ab,kw.
41. (surg* adj1 pain).ti,ab,kw.
42. (("post surg*" or post-surg*) and (pain* or discomfort)).ti,ab,kw.
43. (analgesi* adj4 surg*).ti,ab,kw.
44. (analgesi* adj4 operat*).ti,ab,kw.
45. (sore* or hurt* or ache* or aching or discomfort* or uncomfort* or tender* or throb*).mp. [mp=title, abstract, heading word, table of contents, key concepts, original title, tests & measures, mesh]
46. predictor*.tw,kw.
47. Protective factors/
48. risk assessment/
49. risk factors/
50. (Risk adj factor*).tw,kw.
51. (risk adj assessment*).tw,kw.
52. (protective adj factor*).tw,kw.
53. Prevalence/
54. Prevalence.tw,kw.
55. Incidence/
56. Incidence.tw,kw.
57. Prognosis/
58. Prognos*.tw,kw.
59. correlati*.tw,kw.
60. An?esthetics, Local/
61. An?esthesia, Local/
62. (local adj5 (anesthetic* or anaesthetic* or anesthesia or anaesthesia)).mp.
63. Lidocaine/
64. (lidocaine or lignocaine or xylocaine).mp.
65. Carticaine/
66. (carticain* or articain*).mp.
67. Prilocaine/
68. (prilocain* or citanest* or propitocain* or xylonest).mp.
69. Bupivacaine/
70. (bupivacain* or buvacaina or carbostesin or dolanaest or marcain* or sensorcain* or svedocain*).mp.
71. 1 or 2 or 3 or 4 or 5 or 6
72. 7 or 8 or 9 or 10 or 11 or 12
73. 13 or 14 or 15 or 16 or 17 or 18 or 19 or 20 or 21 or 22 or 23 or 24 or 25 or 26 or 27 or 28 or 29 or 30 or 31 or 32 or 33 or 34 or 35 or 36 or 37 or 38 or 39 or 40 or 41 or 42 or 43 or 44 or 45
74. 46 or 47 or 48 or 49 or 50 or 51 or 52 or 53 or 54 or 55 or 56 or 57 or 58 or 59
75. 60 or 61 or 62 or 63 or 64 or 65 or 66 or 67 or 68 or 69 or 70
76. 71 and 72 and 73 and 74 and 75

Global Health via OVID
1. exp Dentistry/
2. (dental* or dentist*).ti,ab.
3. (oral adj5 surg*).ti,ab.

4. (orthodontic* or pulpotom* or pulpect* or endodont* or "pulp cap*").mp.
5. ((dental or tooth or teeth) adj5 (fill* or restor* or extract* or remov* or "cavity prep*" or caries or carious or decay*)).mp.
6. (root canal and (therap* or treat*)).mp.
7. child/
8. Infant/
9. adolescent/
10. Pediatrics/
11. Dental care for children/
12. (child* or adolescent* or kid or kids or youth* or youngster* or minor or minors or teen* or preteen* or pre-teen* or juvenile* or "young adult*" or "young person" or "young people" or p?ediatric* or student* or pupil or pupils or boy or boys or girl or girls or under 18* or under eighteen* or underage).ti,ab,kw.
13. Pain, Postoperative/
14. (postoperative adj4 pain*).ti,ab,kw.
15. (post-operative adj4 pain*).ti,ab,kw.
16. post-operative-pain*.ti,ab,kw.
17. (post* adj4 pain*).ti,ab,kw.
18. (postoperative adj4 analgesi*).ti,ab,kw.
19. (post-operative adj4 analgesi*).ti,ab,kw.
20. post-operative analgesi*.ti,ab,kw.
21. (post-surgical adj4 pain*).ti,ab,kw.
22. (post surgical adj4 pain*).ti,ab,kw.
23. (pain* adj4 after surg*).ti,ab,kw.
24. (pain* adj4 after operat*).ti,ab,kw.
25. (pain* adj4 follow* operat*).ti,ab,kw.
26. (pain* adj4 follow* surg*).ti,ab,kw.
27. (pain adj1 operati*).ti,ab,kw.
28. (intra adj procedur* adj pain).ti,ab,kw.
29. (intraoperative adj4 pain*).ti,ab,kw.
30. (intra-operative adj4 pain*).ti,ab,kw.
31. intra-operative-pain*.ti,ab,kw.
32. (intraoperative adj4 analgesi*).ti,ab,kw.
33. (intra-operative adj4 analgesi*).ti,ab,kw.
34. intra-operative analgesi*.ti,ab,kw.
35. (intra-surgical adj4 pain*).ti,ab,kw.
36. (intrasurgical adj4 pain*).ti,ab,kw.
37. (pain* adj4 after surg*).ti,ab,kw.
38. (pain* adj4 after operat*).ti,ab,kw.
39. (pain* adj4 follow* operat*).ti,ab,kw.
40. (pain* adj4 follow* surg*).ti,ab,kw.
41. (surg* adj1 pain).ti,ab,kw.
42. (("post surg*" or post-surg*) and (pain* or discomfort)).ti,ab,kw.
43. (analgesi* adj4 surg*).ti,ab,kw.
44. (analgesi* adj4 operat*).ti,ab,kw.
45. (sore* or hurt* or ache* or aching or discomfort* or uncomfort* or tender* or throb*).mp. [mp=abstract, title, original title, broad terms, heading words, identifiers, cabicodes]
46. predictor*.tw,kw.
47. Protective factors/
48. risk assessment/
49. risk factors/
50. (Risk adj factor*).tw,kw.
51. (risk adj assessment*).tw,kw.
52. (protective adj factor*).tw,kw.
53. Prevalence/

54. Prevalence.tw,kw.
55. Incidence/
56. Incidence.tw,kw.
57. Prognosis/
58. Prognos*.tw,kw.
59. correlati*.tw,kw.
60. An?esthetics, Local/
61. An?esthesia, Local/
62. (local adj5 (anesthetic* or anaesthetic* or anesthesia or anaesthesia)).mp.
63. Lidocaine/
64. (lidocaine or lignocaine or xylocaine).mp.
65. Carticaine/
66. (carticain* or articain*).mp.
67. Prilocaine/
68. (prilocain* or citanest* or propitocain* or xylonest).mp.
69. Bupivacaine/
70. (bupivacain* or buvacaina or carbostesin or dolanaest or marcain* or sensorcain* or svedocain*).mp.
71. 1 or 2 or 3 or 4 or 5 or 6
72. 7 or 8 or 9 or 10 or 11 or 12
73. 13 or 14 or 15 or 16 or 17 or 18 or 19 or 20 or 21 or 22 or 23 or 24 or 25 or 26 or 27 or 28 or 29 or 30 or 31 or 32 or 33 or 34 or 35 or 36 or 37 or 38 or 39 or 40 or 41 or 42 or 43 or 44 or 45
74. 46 or 47 or 48 or 49 or 50 or 51 or 52 or 53 or 54 or 55 or 56 or 57 or 58 or 59
75. 60 or 61 or 62 or 63 or 64 or 65 or 66 or 67 or 68 or 69 or 70
76. 71 and 72 and 73 and 74 and 75

PubMed

(((((((((DENTISTRY) OR ((dental* OR dentist*))) OR oral surg*) OR ((orthodontic* OR pulpotom* OR pulpect* OR endodont* OR "pulp cap*"))) OR ((fill* OR restor* OR extract* OR remov* OR "cavity prep*" OR caries OR carious OR decay*))) OR ((root canal and AND (therap* OR treat*))))) AND ((((((child) OR Infant) OR adolescent) OR ((Pediatric* OR paediatric*))) OR Dental care for children) OR (((child* OR adolescent* OR kid OR kids OR youth* OR youngster* OR minor OR minors OR teen* OR preteen* OR pre-teen* OR juvenile* OR "young adult*" OR "young person" OR "young people" OR p?ediatric* OR student* OR pupil OR pupils OR boy OR boys OR girl OR girls OR under 18* OR under eighteen* OR underage))))) AND ((((((((((Pain, Postoperative) OR ((postoperative pain* OR post-operative pain* OR post* AND pain*))) OR ((postoperative analgesi* OR post-operative analgesi* OR post* AND analgesi*))) OR ((postsurgical pain* OR post-surgical pain*))) OR ((pain* AND after surg* OR pain* AND after operat* OR pain* AND follow* AND operat* OR pain* AND follow* AND surg*))) OR Pain, intraoperative) OR ((intraoperative pain* OR intra-operative pain* OR intra* AND pain*))) OR ((intraoperative analgesi* OR intra-operative analgesi* OR intra* AND analgesi*))) OR ((intrasurgical pain* OR intra-surgical pain*))) OR ((sore* OR hurt* OR ache* OR aching OR discomfort* OR uncomfort* OR tender* OR throb*)))) AND ((((((((predictor*) OR Protective factors) OR risk assessment) OR risk factors) OR Prevalence) OR Incidence) OR Prognos*) OR correlati*)) AND ((((local anesthetic* OR local anaesthetic* OR local anesthesia OR local anaesthesia))) OR ((lidocain* OR lignocain* OR xylocain* OR carticain* OR articain* OR prilocain* OR citanest* OR propitocain* OR xylonest OR bupivacain* OR buvacaina OR carbostesin OR dolanaest OR marcain* OR sensorcain* OR svedocain*)))

Scopus

(TITLE-ABS-KEY ("dental treatment" OR "dentistry" OR "dental care" OR "dental therapy*") AND TITLE-ABS-KEY (child* OR adolescent* OR kid* OR youth* OR

youngster* OR minors OR teen* OR preteen* OR pre-teen* OR juvenile* OR "young adult*" OR "young person" OR "young people" OR p?ediatric* OR student* OR pupil* OR boy* OR girl*) AND TITLE-ABS-KEY (pain OR sore* OR hurt* OR ache* OR aching OR discomfort* OR uncomfort* OR tender* OR throb*) AND TITLE-ABS-KEY (predictor* OR "Protective factor*" OR "risk assessment*" OR "risk factor*" OR prevalence OR incidence OR prognos* OR correlati*) AND TITLE-ABS-KEY ("Local An?esthetics" OR "Local An?esthesia" OR lidocaine OR lignocaine OR xylocaine OR carticain* OR articain* OR prilocain* OR citanest* OR propitocain* OR xylenes OR bupivacain* OR bupivacaina OR carbostesin OR solanales OR marcain* OR sensorcain*))

SciELO (Web of Science)
26 #24 AND #23 AND #18 AND #7 AND #6
Refined by: [excluding] WEB OF SCIENCE CATEGORIES: (ANESTHESIOLOGY OR CRITICAL CARE MEDICINE OR PERIPHERAL VASCULAR DISEASE OR MEDICINE GENERAL INTERNAL OR MEDICINE RESEARCH EXPERIMENTAL OR PHARMACOLOGY PHARMACY OR OBSTETRICS GYNECOLOGY OR PUBLIC ENVIRONMENTAL OCCUPATIONAL HEALTH OR PEDIATRICS OR OTORHINOLARYNGOLOGY OR TOXICOLOGY)
Indexes=SCI-EXPANDED, SSCI, A&HCI, CPCI-S, CPCI-SSH, ESCI Timespan=All years
# 25 #24 AND #23 AND #18 AND #7 AND #6
Indexes=SCI-EXPANDED, SSCI, A&HCI, CPCI-S, CPCI-SSH, ESCI Timespan=All years
# 24 ALL=(local an?esthetic* or local an?esthesia)
Indexes=SCI-EXPANDED, SSCI, A&HCI, CPCI-S, CPCI-SSH, ESCI Timespan=All years
# 23 #22 OR #21 OR #20 OR #19
Indexes=SCI-EXPANDED, SSCI, A&HCI, CPCI-S, CPCI-SSH, ESCI Timespan=All years
# 22 ALL FIELDS: (Incidence)
Indexes=SCI-EXPANDED, SSCI, A&HCI, CPCI-S, CPCI-SSH, ESCI Timespan=All years
# 21 ALL FIELDS: (Prevalence)
Indexes=SCI-EXPANDED, SSCI, A&HCI, CPCI-S, CPCI-SSH, ESCI Timespan=All years
# 20 ALL FIELDS: (risk factors)
Indexes=SCI-EXPANDED, SSCI, A&HCI, CPCI-S, CPCI-SSH, ESCI Timespan=All years
# 19 ALL FIELDS: (predictor*)
Indexes=SCI-EXPANDED, SSCI, A&HCI, CPCI-S, CPCI-SSH, ESCI Timespan=All years
# 18 #17 OR #16 OR #15 OR #14 OR #13 OR #12 OR #11 OR #10 OR #9 OR #8
Indexes=SCI-EXPANDED, SSCI, A&HCI, CPCI-S, CPCI-SSH, ESCI Timespan=All years
# 17 ALL FIELDS: (sore* or hurt* or ache* or aching or discomfort* or uncomfort* or tender* or throb*)
Indexes=SCI-EXPANDED, SSCI, A&HCI, CPCI-S, CPCI-SSH, ESCI Timespan=All years
# 16 ALL FIELDS: (intrasurgical pain* or intra-surgical pain*)
Indexes=SCI-EXPANDED, SSCI, A&HCI, CPCI-S, CPCI-SSH, ESCI Timespan=All years
# 15 ALL FIELDS: (intraoperative analgesi* or intra-operative analgesi* or intra* analgesi*)
Indexes=SCI-EXPANDED, SSCI, A&HCI, CPCI-S, CPCI-SSH, ESCI Timespan=All years
# 14 ALL FIELDS: (intraoperative pain* or intra-operative pain* or intra* pain*)
Indexes=SCI-EXPANDED, SSCI, A&HCI, CPCI-S, CPCI-SSH, ESCI Timespan=All years
# 12 ALL FIELDS: (pain* after surg* or pain* after operat* or pain* follow* operat* or pain* follow* surg*)
Indexes=SCI-EXPANDED, SSCI, A&HCI, CPCI-S, CPCI-SSH, ESCI Timespan=All years
# 11 ALL FIELDS: (postsurgical pain* or post-surgical pain*)
Indexes=SCI-EXPANDED, SSCI, A&HCI, CPCI-S, CPCI-SSH, ESCI Timespan=All years
# 10 ALL FIELDS: (postoperative analgesi* or post-operative analgesi* or post* analgesi*)
Indexes=SCI-EXPANDED, SSCI, A&HCI, CPCI-S, CPCI-SSH, ESCI Timespan=All years
# 9 ALL FIELDS: (postoperative pain* or post-operative pain* or post* pain*)
Indexes=SCI-EXPANDED, SSCI, A&HCI, CPCI-S, CPCI-SSH, ESCI Timespan=All years

# 8 ALL FIELDS: (Pain, Postoperative)
Indexes=SCI-EXPANDED, SSCI, A&HCI, CPCI-S, CPCI-SSH, ESCI Timespan=All years
# 7 ALL=(child* or adolescent* or kid or kids or youth* or young* or minor or minors or teen* or preteen* or pre-teen* or juvenile* or p?ediatric*)
Indexes=SCI-EXPANDED, SSCI, A&HCI, CPCI-S, CPCI-SSH, ESCI Timespan=All years
# 6 #5 OR #4 OR #3 OR #2 OR #1
Indexes=SCI-EXPANDED, SSCI, A&HCI, CPCI-S, CPCI-SSH, ESCI Timespan=All years
# 5 ALL FIELDS: (root canal and (therap* or treat*))
Indexes=SCI-EXPANDED, SSCI, A&HCI, CPCI-S, CPCI-SSH, ESCI Timespan=All years
# 4 ALL FIELDS: (fill* or restor* or extract* or remov* or "cavity prep*" or caries or carious or decay*)
Indexes=SCI-EXPANDED, SSCI, A&HCI, CPCI-S, CPCI-SSH, ESCI Timespan=All years
# 3 ALL FIELDS: (orthodontic* or pulpotom* or pulpect* or endodont* or "pulp cap*")
Indexes=SCI-EXPANDED, SSCI, A&HCI, CPCI-S, CPCI-SSH, ESCI Timespan=All years
# 2 ALL FIELDS: (oral surg*)
Indexes=SCI-EXPANDED, SSCI, A&HCI, CPCI-S, CPCI-SSH, ESCI Timespan=All years
# 1 ALL FIELDS: (DENTISTRY)
Indexes=SCI-EXPANDED, SSCI, A&HCI, CPCI-S, CPCI-SSH, ESCI Timespan=All years
